# Exploring how greenspace programmes might be effective in supporting people with problem substance use: a realist interview study

**DOI:** 10.1186/s12889-022-14063-2

**Published:** 2022-09-01

**Authors:** Wendy Masterton, Tessa Parkes, Hannah Carver, Kirsty J. Park

**Affiliations:** 1 Salvation Army Centre for Addiction Services and Research, Faculty of Social Sciences, University of Stirling, Stirling, FK9 4LA Scotland, UK; 2 Biological and Environmental Sciences, University of Stirling, Stirling, FK9 4LA Scotland, UK

**Keywords:** Greenspace, Natured-based interventions, Green care, Substance use, Mental health

## Abstract

**Background:**

Greenspace programmes are health projects run outside in nature, typically with the aim of improving mental health. Research suggests that programmes may also be effective in supporting people with problem substance use (PSU), but there is limited understanding of the key components that make them successful for this client group. Previous work has claimed that a realist-informed intervention framework for greenspace programmes for mental health could be transferable to programmes that support people with PSU, and that this could provide insight into the causal processes within programmes. However, this claim is yet to be explored in depth. The aim of this study was to further test, refine, and consolidate the proposed framework to show how greenspace programmes could support people with PSU.

**Methods:**

Using a realist approach, 17 participants (8 programme staff; 9 wider stakeholders) were interviewed about contexts, mechanisms, and outcomes (CMOs) relative to greenspace programmes for mental health and PSU. Semi-structured interviews were used since they facilitated exploration of the proposed framework but were flexible enough to allow identification of new CMOs for framework refinement. Interviews were audio-recorded, fully transcribed, and analysed inductively and deductively against the proposed framework.

**Results:**

Findings supported the proposed framework and indicated that greenspace programmes support people with poor mental health and PSU due to: feelings of escape; space to reflect; physical activity; self-efficacy; feelings of purpose; relationships; and shared experiences. However, data showed that programmes must also consider: explicit intervention focus to ensure adequate support for clients; existing challenges with funding and stakeholder buy-in; and the impact of COVID-19. Findings allowed development of a refined framework that shows how greenspace programmes can support people with PSU.

**Conclusion:**

The findings of this project are theoretically novel and have practical relevance for those designing greenspace programmes by providing recommendations on how to optimise, tailor, and implement future interventions. Findings could be particularly relevant for academic researchers, multidisciplinary health professionals, and for those working in the third sector, developing and delivering greenspace programmes for people to improve their mental health and to support them with PSU.

**Supplementary Information:**

The online version contains supplementary material available at 10.1186/s12889-022-14063-2.

## Background

Evidence of the link between greenspace and positive mental health is rapidly growing. To achieve desired mental health outcomes, research suggests that exposure to greenspace environments is not enough, and there must also be a planned intervention to encourage use of the space [1, 2]. Greenspace programmes are a type of targeted health intervention implemented in a variety of green settings like public parks, woodlands, wilderness, gardens, farms, and allotments, among others [3–5]. A wide range of different activities may be employed as part of these programmes such as: gardening or horticulture; organised walks for wellbeing; forest walks and forest bathing; wilderness or adventure programmes; outdoor woodland learning; nature-based mindfulness; conservation activities; and care farming [3, 4, 6]. Previous systematic reviews of greenspace programmes for mental health have provided some evidence of efficacy [6–10]. Despite this, without knowing the components, processes, and influences within programmes, it is difficult to understand why the interventions work, and how best to replicate them. Other studies have provided more detailed accounts of the mechanisms of change by which engagement with nature impacts physical and mental health [3, 4, 11], but continued exploration of how different contexts are likely to facilitate different mechanisms and outcomes is important, as what ‘works’ in one setting might not ‘work’ in a different one.

To address this gap, Masterton et al. [12] conducted a realist review to synthesise the international evidence for greenspace programmes for mental health and explore how these work, why they work, for whom, how context influences mechanisms, and how mechanisms lead to outcomes. Realist methodology holds that the outcomes related to complex intervention programmes, such as changes in behaviour, are directly caused by underlying generative mechanisms, described as the invisible elements of reasoning and reaction [13]. For these mechanisms to happen, they must be activated in the right contexts. Contexts can be individual, interpersonal, organisational, or institutional factors [13]. Such causal relationships between contexts, mechanisms, and outcomes are referred to as context-mechanism-outcome configurations (CMOcs), and these CMOcs are described as the ‘programme theories’ of why an intervention works. Masterton et al. [12] identified and refined seven programme theories (Escape and Getting Away; Space to Reflect; Physical Activity; Self-Efficacy; Having a Purpose; Relationships with Facilitators; and Shared Experiences) to show how greenspace programmes work under three proposed themes of Nature, Individual Self, and Social Self. The programme theories allowed an understanding of greenspace programmes in general and helped explain how optimum mental health outcomes, such as decreased stress, improved mood and self-esteem, and improved social cohesion, among others, could be achieved.

Some emerging evidence has also shown that greenspace programmes appear to be effective for supporting people with problem substance use (PSU) [14–17]. Similar mechanisms that were identified in Masterton et al. [12], such as increased self-efficacy, feelings of purpose, and improved relationships, are core components of successful substance use interventions [18, 19]. If these mechanisms are activated within greenspace programmes, it is therefore feasible that these types of interventions could be effective in supporting people with PSU too. To explore this, the aim of this study was to use a realist interview approach to further test, refine, and consolidate the CMOcs identified in Masterton et al.’s framework [12] for use on greenspace programmes that support people with PSU.

## Methods

All study methods were performed in accordance with the relevant guidelines and regulations. Ethical approval for the study was granted by the General University Ethics Panel (GUEP) at the University of Stirling (GUEP (19 20) 959). Written informed consent from participants was granted before each interview, as described below.

Realist interviews typically start as exploratory interviews, before moving onto refinement and consolidation of identified programme theories [20, 21]. Since Masterton et al.’s original framework [12] provided initial programme theories for testing, this reduced the need for numerous exploratory interviews and, instead, allowed testing and refinement of programme theories to happen straight away, alongside more exploratory questions. The interview process was therefore split into two stages. The first stage allowed testing and refinement of programme theories, and the second stage allowed consolidation. The interview schedule was refined for stage two in response to stage one data (see Additional file 1 for interview schedules). Concurrent analysis of the data showed that, by the fifth interview in stage two, no new themes were being identified relating to programme theories, so interviews were ceased at this point.

### Stage one recruitment and participants

All participants were identified through existing networks of the research team, and purposive sampling was used to select individuals based on gender, role, and organisation to try to ensure the sample reflected a wide range of views and experiences. Participants were recruited from Scotland (*n* = 5), other United Kingdom (UK) nations (*n* = 5), and international organisations (*n* = 2). This was important given the international focus of the previous realist work [12], and global interest in greenspace interventions. Two potential participants declined involvement due to their increased workload as a result of the pandemic. To provide the necessary depth of information, two categories of participants were interviewed. The first category were staff that worked directly on greenspace programmes with people who use drugs and/or alcohol (*n* = 6). Two staff worked on wilderness-based programmes, three staff worked on garden-based programmes, and one staff member worked on both rural and urban conservation settings. By interviewing staff from programmes that used different greenspace settings, this ensured the framework was tested and refined using data from a range of programmes. This was important as it increased the framework’s transferability across programme types.

The second category of participants interviewed were wider stakeholders whose work was directly linked to greenspace programmes for substance use support but did not work on the programmes themselves (*n* = 6). Five stakeholders were academic researchers whose previous work on projects relating to greenspace programmes allowed valuable insight into the proposed CMOcs, as well as identifying refinements and additions to the programme theories. One stakeholder was a National Health Service (NHS) practitioner who had previously been involved in supporting clients onto greenspace programmes and who still had an interest in advocating for the health benefits of time spent in nature. No clients were included in the sample as, due to COVID-19, the majority of greenspace programmes were not operational/accepting visitors during the period of data collection. An initial recruitment email was sent to potential participants letting them know about the study and aims. Interested individuals were asked to respond to the email, upon which a participant information sheet and consent form were emailed to them. Consent forms were stored on a secure MS Teams channel. All participants were assured that participation was voluntary, and they could withdraw at any time without giving a reason.

### Stage one interview process

Twelve interviews lasting an hour on average were conducted electronically and audio recorded. While all interview questions were directly related to the CMOcs identified in Masterton et al.’s framework [12], the interview schedule (Additional file 1) remained broad enough to be exploratory where necessary and allowed identification of new contexts, mechanisms, and outcomes.

### Stage one data analysis

Data were transcribed in full, analysed, and coded thematically in NVivo 12 by the lead author, with this process checked by all additional authors. This provided opportunities for discussion on anything that was unclear or could have different interpretations, and therefore enhanced rigour [22]. There is sparse evidence of how best to use NVivo within realist research, so to try to ensure transparency and best practice, guidance was taken from two key papers, Gilmore et al. [23] and Dalkin et al. [24]. These papers discuss how coding in NVivo is beneficial as it allows inductive approaches (codes emerging from the data) [25] and deductive approaches (codes developed from the research question) to be used [26]. This facilitated testing the data against the proposed programme theories, but also allowed identification of new CMOcs. Transcripts were read in full and then coded line by line. The staff interviews were analysed and coded first which allowed the first iteration of programme theory refinement. The stakeholder interviews were analysed and coded second allowing further refinement prior to the stage two consolidation interviews. Finally, each transcript was re-read for completeness to ensure that the final framework was inclusive of all major themes. Memo boxes were used throughout data analysis as a way of reflecting on the data and recording refinements between stages one and two.

### Stage two recruitment and participants

All participants were different to those in stage one and had not been approached previously, but the recruitment process was the same. As in stage one, all participants were identified through existing networks, and purposive sampling was used to select individuals whose expertise would provide insight into the proposed programme theories. There were no potential participants who declined to take part in this stage. As before, two categories of participants were interviewed. Relating to the staff that worked directly on greenspace programmes with people who use drugs and/or alcohol (*n* = 2), one staff member worked on both rural and urban conservation settings, and one staff member worked on a garden-based programme. The second category was again wider stakeholders whose work was directly linked to greenspace programmes for substance use support but did not work on the programmes themselves (*n* = 3). In stage one, five academic researchers were interviewed, but only one practitioner; so, the decision was made to prioritise recruitment of practitioners in stage two, rather than academic researchers. Two stakeholders were NHS practitioners who had experience with green prescribing, and one stakeholder worked in the third sector and had experience with green prescribing and greenspace programme development. Three participants were from Scotland, one was from another UK nation, and one was from an international organisation.

### Stage two interview process and data analysis

Five interviews lasting an hour on average were conducted electronically and were audio recorded. Data analysis occurred through the same process as in stage one. However, in this final stage of the realist interview, the findings refine and consolidate the programme theory [20, 21]. This final stage is important as it can facilitate: better understanding of proposed mechanisms, or identification of new mechanisms; better understanding of key contextual factors; or a more refined understanding of the patterns of outcomes resulting from the interaction of context and mechanism. Again, NVivo memo boxes were used to keep track of the refinements and subsequent consolidation of the programme theories in this stage, and final CMOcs were written down and discussed with the full research team to ensure agreement.

## Results

### Stage one interviews: staff and stakeholder findings

As shown in Table 1, findings are presented under the programme theory headings proposed in Masterton et al.’s original framework [12], as well as under newly identified programme theories from this study’s data. Participant details and their IDs which are used to attribute direct quotes are shown below in Table 2.

**Table 1:** Programme theory themes and headings informed by Masterton et al. [12] and this study’s data

Programme theory theme	Programme theory heading
Nature	1. Escape and getting away 2. Space to reflect
Individual self	3. Physical activity 4. Self-efficacy 5. Having a purpose
Social self	6. Relationships with facilitators 7. Increased communication through shared experiences 8. Reduced isolation
Macro-level	9. COVID-19 impact
Meso-level	10. Intervention approach 11. Stakeholder buy-in

**Table 2:** Participant details and pseudonyms

Participant role	Staff/ stakeholder	Setting of programme	Location	Pseudonym
Manager	Staff	Wilderness	Scotland	Rob
Support worker	Staff	Wilderness	Scotland	Malcolm
Manager	Staff	Garden	Scotland	Gerry
Manager	Staff	Conservation	UK	Michael
Volunteer	Staff	Garden	Scotland	Alan
Manager	Staff	Garden	Scotland	Jess
Director of research institute	Stakeholder	N/A	International	Gillian
Research fellow	Stakeholder	N/A	International	Sarah
Research fellow	Stakeholder	N/A	UK	Hayley
Research fellow	Stakeholder	N/A	UK	Jack
Research fellow	Stakeholder	N/A	UK	Laura
NHS practitioner	Stakeholder	N/A	Scotland	Ross

### Programme theory theme: nature

#### Programme theory one: escape and getting away

Ease of access appeared to be a more prevalent context than initially identified in Masterton et al.’s framework [12]. One staff member spoke about how it was helpful having their programme near city centre pharmacies visited by people who used drugs. This was compared to another location situated further from the city centre where they have had “*nowhere near the success*” (Alan, Staff). As well as proximity, stakeholder data discussed “*issues of uneven surfaces, slippery surfaces, […] lighting […] that feeling of safety”* (Laura, Stakeholder) as impacting access. The greenspace was described as needing to be of quality. The subjectivity of what ‘quality’ means relative to greenspace was discussed at length, and there was general agreement that when greenspace has higher biodiversity and various stimuli such as bird song, and is free of litter, graffiti, and other vandalism, then it is more likely people will view it as high quality and want to spend time there. Relating to individual-level contextual factors, clients’ previous experience of nature was said to influence programme success:

*Clients not being used to the outdoors, you know. You can see them in the first couple of meetings […] looking at you as if “listen mate there is no way in this world you are getting me out there camping for ten days, are you mad?”.* (Malcolm, Staff).

The key mechanism in this programme theory was identified by both staff and stakeholders as a feeling of escape and getting away. This mechanism appeared to be particularly key for people who use substances:

*It* [the programme] *gets them away from the sort of rat race that they are stuck in. A lot of the guys that we work with, they are in the house, and then they are out the house, they are down to the chemist getting their prescription, and then they are either going and scoring, or just going straight back home.* (Alan, Staff).

Other identified mechanisms were: feelings of calm; feelings of being ‘in the moment’, and reduced rumination. Interviewees also described participants as experiencing spiritual feelings and feeling *“blown away”* (Rob, Staff), with one explaining that *“you find that most clients, when they are out there, it kind of takes their breath away*”. (Malcolm, Staff). Interview data showed connection to nature was another mechanism in this programme theory, and this was a refinement from Masterton et al.’s original framework [12] where connection to nature was identified as an outcome. It could be argued that a connection to nature might be either mechanism or outcome, depending on the CMOc. However, literature typically depicts mechanisms as being unmeasurable and hidden [13, 27], so, in this line of thinking, it is likely that connection to nature is a mechanism. A connection to nature was said to take place in all types of greenspace programmes suggesting that the quality of the greenspace was perhaps more important than the type of greenspace programme activity in facilitating this connection to nature. Outcomes in this programme theory were identified as *“improved mental wellbeing”* (Laura, Stakeholder) and *“reduced stress and anxiety”* (Sarah, Stakeholder), with interviewees agreeing that outcomes were wide ranging depending on the individual.

#### Programme theory two: space to reflect

Programmes were described as most successful when clients were able to attend for longer periods of time. Further, the physical space on the greenspace programme was described as an important environmental context. One staff member discussed how, when a person is struggling with issues such as PSU, they can “*feel trapped and very enclosed”* (Gerry, Staff)*,* and the greenspace programme can provide physical space to mitigate this. A refinement seen in stakeholder data was that the neutrality of the space was also important, with greenspace being *“a non-threatening environment”* (Gillian, Stakeholder), and “*non-institutional”* (Hayley, Stakeholder). The central mechanism in this programme theory was said to be the feeling of no longer being confined and boxed in, and having the physical and mental space to reflect:

*If you are embroiled in the world of substance use […] being able to get some distance and some perspective on your life is really, really good. […] Looking at the mountains, looking at the trees, hearing the rivers, connecting with nature, with being outside. You are sort of separating from your own agenda and your own issues.* (Gerry, Staff).

The use of metaphors was also linked to reflection, for example when reflecting on aspects of self-care:

*When we work with people with addictions, for example, it is very important to give them a sick plant […] and don’t tell the participant what to do with the plant, just ask the participant questions, what will you do? Do you want to water it? Do you want to prune it? Does it need nutrients? Do you need to change the soil? It’s the best way to make obvious what might not be obvious to them.* (Gillian, Stakeholder).

Although the original framework [12] identified a desire to change as being the main outcome for this programme theory, interview data in this study highlighted that a more accurate outcome was increased sharing and ‘opening up’ by clients about their lives, challenges, and problems:

*One of the things about natural environments as a setting for health promotion is that it’s a sort of neutral space, a non-institutional space, which allows people to think about things differently, and have different types of conversations, than they would do in a therapist’s room or a doctor’s setting.* (Hayley, Stakeholder).

### Programme theory theme: individual self

#### Programme theory three: physical activity

The availability of existing resources, such as kit and equipment, was described as necessary to facilitate activities. For example, having the appropriate clothes and footwear for clients to stay warm and dry was described as crucial, as was having the right tools to safely undertake activities. Availability of experienced staff was also considered to be imperative for programme success, particularly to encourage people onto programmes:

*Just the existence of staff in the first place. […] It sounds obvious, but we work on quite a lot of projects where there can be a bit of a “build it and they will come” kind of attitude.* (Michael, Staff).

However, even with the right resources, weather was described as a further context to consider and, where necessary, adapt to by providing shelter and warmth. A contextual refinement taken from stakeholder interviews was that time commitment likely influenced physical activity outcomes because “*you’ve got to spend enough time there for it to be beneficial”* (Hayley, Stakeholder). Interviewees spoke about the need for a range of different activities to facilitate an enjoyment of activities, as this enjoyment was an important mechanism. Increased physical activity, improved mental wellbeing, and improved physical health were all confirmed by the data as outcomes, with the link between physical and mental health outcomes described as a “*circle of impact”* (Sarah, Stakeholder).

#### Programme theory four: self-efficacy

As in the theory above, availability of experienced facilitators was considered an essential context for programme success. With facilitators present to guide and support clients, this was said to aid clients enhance their skillset. However, the mechanism of learning new skills could also be about “*reconnecting with old skills”* (Gerry, Staff) that had not been used in a long time. An example was given of a client who had the opportunity to use skills he had learnt when he was younger:

*He was the one who led the group because he had all the skill and knowledge from when he was a wee lad. This guy is almost in his fifties, but he was lost for 25, 30 years in the world of drug use.* (Gerry, Staff).

Learning skills could also be related to new psychological skills, for example through emotional regulation or problem-solving. Regardless of the type of skill, an enhanced skillset was described as leading to increased self-esteem:

*The vehicle of going out and camping, walking, and putting up tents and chopping up sticks and making a fire and catching, whatever it might be, is just the vehicle. Through that, what the person gets is an increased confidence […] they find that they can do things, which then loops back on itself in a kind of “okay I’m good enough” sort of way.* (Ross, Stakeholder).

Interviewees also discussed how new skills learnt on programmes could be transferable to clients’ lives outside the programme and help them cope better with challenges encountered in their day-to-day lives. Indeed, one interviewee said that an important part of the programme was to explore *“how do we cope without relying on alcohol and chemicals to deal with life, because life can be really, really tough”* (Gerry, Staff).

#### Programme theory five: having a purpose

Interviewees referred to the routine of the programme as an important context, but this did not imply programmes should be rigid and inflexible and, instead, was more closely linked to being a reliable presence and something constant in a client’s life:

*You have people who have been in the house for six months, and actually getting up in the morning and putting on their socks was considered a real benefit to their day.* (Jack, Stakeholder).

The other context identified through the data relative to this programme theory was that the programme had to be person-centred. Although greenspace programmes appear to activate similar mechanisms across a range of people, regardless of programme activity, interviewees discussed how the activities themselves should still be individualised. If a programme is structured, and provides person-centred support, this was said to facilitate the mechanisms of purpose and achievement:

*Purpose is one of the big things. It’s to give them some kind of “something” they see themselves as getting into, and it’s fulfilling a need that they didn’t have before.* (Malcolm, Staff).

Feelings of purpose and achievement were said to result in outcomes of improved self-esteem and confidence which could subsequently impact other areas of life such as enabling future planning.

### Programme theory theme: social self

#### Programme theory six: relationships with facilitators

For clients to buy into greenspace programmes, one of the key contexts was described as needing a ‘doing with’, rather than ‘doing for’, culture:

*If we stand on the side-lines and don’t get involved, then there is not so much room for communication as you’ve still got that sort of official role that you are playing. But when you get your sleeves up and start getting involved with them, you start chatting about this, that, and the next thing, and then they suddenly feel a bit more comfortable.* (Alan, Staff)*.*

Having a trauma-informed culture of care was also described as essential so that the programme staff can better understand the challenges that clients may have in forming relationships:

*We are talking about a group of people who tend to have had large amounts of trauma and adversity in their background, and use of substances is a management of that. […] If you are working with somebody who has never, I mean genuinely in their life, never really had anybody who has believed in them, or thought they were worth anything, or taken the time to pay any attention to them, it might take years and years and years of you believing in them until they believe in themselves.* (Ross, Stakeholder)*.*

A contextual refinement in this study’s findings was that stakeholders identified the need for diversity within the facilitating/staff team. It was proposed that there should be a mix of genders, as well as ethnicities:

*If you felt you don’t belong, you don’t want to go, do you? It’s very difficult to build those relationships if you don’t have those common languages or common points of reference and support.* (Hayley, Stakeholder).

The above contexts were said to facilitate reductions in power imbalances between facilitators and clients. One interviewee described this mechanism as the *“level playing field”* (Rob, Staff). Further, trust and feelings of safety were deemed by interviewees as essential for building relationships, with one staff member describing these mechanisms as “*the main thing”* (Malcolm, Staff) in the success of programmes. Stakeholders also identified increased communication between facilitator and client as an additional mechanism. Engagement and continued buy-in by clients were confirmed through the data as the main outcomes in this programme theory. One interviewee explained that continued buy-in was important because:

*People who have completed a course are much more likely to continue to engage with you and come back.* (Malcolm, Staff).

#### Programme theory seven (revised): increased communication through shared experiences

The programme theory of ‘Shared Experiences’ seen in Masterton et al. [12] has been split into two separate programme theories as interview data from this study indicated that the original theory was too broad. The title of the first revised programme theory is ‘Increased Communication through Shared Experiences’. According to the data, greenspace programmes provide an enabling environment for communication, in comparison to medicalised, clinical environments. However, interviewees spoke about the need to have trained facilitators present to allow clients from different, often complex, backgrounds to navigate their interactions:

*People can be quite complex, so it’s good to have somebody that has got some training, and has got some competencies around just supporting people, and being safe, and fair, and honest with people who may have different challenges going on in their lives, particularly when you mix people together*. (Gerry, Staff).

The engagement of others was also deemed an important contextual factor as when clients saw others engaging with the programme activities they often “*followed suit and did the same thing”* (Rob, Staff). Shared experiences *“give the opportunity for participants to communicate and work on a project together”* (Sarah, Stakeholder)*,* which enables rapport to be built. This mechanism of increased communication through shared experiences was said to lead to the outcome of improvements in peer and other relationships. One interviewee proposed that improved relationships were the most important outcomes on programmes, over and above substance use-related outcomes:

*What you are really doing is building connections and building relationships to the point where, if you do it long enough, the substance use might take care of itself, because other things have taken its place.* (Ross, Stakeholder).

#### Programme theory eight (revised): reduced isolation

The second revised theory from the original ‘Shared Experiences’ programme theory is titled ‘Reduced Isolation’. The contexts related to this programme theory were shown through interview data to be the same as the contexts in the programme theory above, which is unsurprising since they were originally the same theory. The central mechanisms were said to be increased understanding of others and reduced stigma:

*We’ve got guys that go down there who have got a lot of substance issues, and they will be working beside somebody from the community that has maybe got a bit of a stigmatised view towards that. As soon as they are getting their hands dirty and they are working away, that all falls away. So, it opens up the ground for discussions there, and for people to actually start looking at the positives, rather than the differences.* (Alan, Staff).

Increased understanding of others and a reduction in stigma reportedly allows clients to integrate back into their communities in a way they had not before. One staff member explained that clients report how they are once again “*part of the world, being seen, being heard, feeling like a person”* (Jess, Staff).

### Programme theory theme: macro-level

#### Programme theory nine (new): COVID-19 impact

Realist research proposes programme theories of the social world as it exists in that moment, and COVID-19 was identified as a macro-level contextual factor that had affected all greenspace programmes over the course of the study. Indeed, the context of the pandemic forced changes and many services had to either shut or adjust. One staff member explained that *“the whole ethos of the organisation has been challenged”* (Malcolm, Staff), and many programmes were reportedly unable to run or provide the same level of support to clients as “*you just can’t build relationships over Zoom, they become artificial”* (Rob, Staff). Interviewees said that the inability to provide the same support negatively affected client trust in services with one explaining that they would try to encourage clients back to programmes to then have to inform them that programmes were cancelled again:

*A guy this morning, he was quite nervous about going away, but he got around to it, and I think he’s quite looking forward to it, and then this morning I’m getting his fares and all that sorted, just giving him reassurance, going over certain guidelines and stuff and then having to phone him up and telling him that it’s cancelled.* (Malcolm, Staff).

Some also discussed how the pandemic had created feelings of hopelessness among clients due to losing their support system. While staff described their efforts in responding to challenges posed by the pandemic, and keeping in touch with clients, the mechanisms of reduced trust and reduced hope were said to lead to decreased mental wellbeing and reduced engagement:

*95% of young people who are classed as vulnerable have disappeared and, by that, I mean they have stopped engaging with services. So, either the service has stopped, or whatever services that kept going, they have lost those people, they have just no contact with them.* (Rob, Staff)*.*

### Programme theory theme: Meso-level

#### Programme theory ten (new): intervention approach

Interviewees discussed how programmes could be developed with a recovery focus, prevention focus, or with the aim of providing holistic support not specific to PSU. Programme focus must therefore be explicit to identify for whom the programme is intended, and what outcomes are deemed desirable, feasible, and attainable for clients. By deciphering programme focus, this reportedly ensures a person-centred approach which then facilitates the mechanism of feeling supported:

*If there is a lack of support, and an expectation-delivery gap, that can have an independent effect in itself, never mind the effectiveness on the programme.* (Hayley, Stakeholder).

According to the data, outcome goals should also be individualised, based on the context of the programme, to be readily accepted by clients. One interviewee said that, in their opinion, focusing explicitly on reducing substance use was “*missing the point”* (Ross). For example, in holistic programmes that provide multilayers of support, clients may not feel defined by their substance use for the first time, if the programme outcomes are not specific to reductions in use. Therefore, relying on symptom outcome measures, such as quantifying substance use, may not provide adequate information:

*A whole load of other stuff might get missed… actually what gave rise to the person needing to have a relationship with substances in the first place […] it would be a red herring or a misnomer to call it a substance use intervention, it’s got nothing to do with that, it’s a relationship intervention, it’s a connection intervention, a side product of which might be that the person may, in time, be less dependent on substances.* (Ross, Stakeholder).

#### Programme theory eleven (new): stakeholder buy-in

To encourage stakeholder buy-in, funding availability was identified in the data as a macro-level contextual factor:

*It’s the obvious thing to say every time, but it is a huge barrier, not just the lack of quality funding, but it’s just very short term […] if you go to them and say we are running this programme for the next six weeks, they are not really that interested, because by the time they’ve actually spoken to people, and started to refer people into it, it’s going to be gone again.* (Michael, Staff).

Even with funding, another contextual factor that influenced buy-in was that those running the programmes must ensure funders, or other key stakeholders, such as those referring clients onto programmes, understand their purpose:

*There is absolutely a job on the part of whoever, me, you, to educate funders about what it is that they are actually funding.* (Ross, Stakeholder).

Through the interviews it became clear that that existing beliefs about programmes could also have an impact. If GPs, funders, or other relevant stakeholders, already had awareness about the benefits of greenspace, they were more likely to have positive feelings towards them. On the other hand, if they had no experience of greenspace programmes, “*they are very much less likely to refer”* (Jack, Stakeholder). Within these contexts, stakeholders are more likely to feel like the programme is worthwhile, and such “buy-in” was described as the key mechanism in this programme theory. Two specific buy-in outcomes were increased onward referrals onto programmes by GPs, and increased availability of programmes due to continued funding.

### Stage two interviews: consolidation

The stage two interviews provided data to consolidate the final programme theories for greenspace programmes that support people with PSU. Stage two participant details and their pseudonyms are shown below in Table 3.

**Table 3:** Stage two participant details and pseudonyms

Participant role	Staff/stakeholder	Location	Pseudonym
Programme manager	Staff	UK	Harry
Third sector practitioner	Stakeholder	Scotland	Gemma
NHS practitioner	Stakeholder	Scotland	Richard
NHS practitioner	Stakeholder	Scotland	Annie
Programme manager	Staff	International	Beth

### Programme theory theme: nature

#### Programme theory one: escape and getting away

Ease of access, quality greenspace, and previous experience of nature were all confirmed to be the main contextual factors in this programme theory. Interviewees confirmed that higher levels of biodiversity and clean, accessible spaces represented quality greenspace, in their opinion. Feelings of ‘being away’, feelings of being ‘in the moment’, reduced rumination, and spiritual feelings of awe were all confirmed as mechanisms. Supporting stage one data, connection to nature was established as a mechanism. Through these mechanisms, outcomes were confirmed to be improved mental wellbeing and reduced stress.

#### Programme theory two: space to reflect

The physical space provided by the greenspace environment was confirmed by the data as a necessary contextual factor, and the longer a client took part in the programme the more likely they were to benefit. The refinement of the space being neutral and non-clinical was supported in the consolidation interviews, with greenspace described as *“a very non-threatening, supportive place […] in comparison with clinical appointments”* (Beth, Staff). The key mechanisms in this programme theory were confirmed as the feeling of not being ‘boxed in’ and having space for reflection. Consolidation interviews confirmed that the outcomes relating to this programme theory were increased opening up and discussion by the client. One interviewee spoke about how some clients felt frustrated during traditional treatment approaches which often consist of repetitive conversations:

*The clinical model is almost a self-fulfilling prophecy […] it’s “right how much have you been using? How much have you been drinking?” Very problem saturated still. Whereas I think the feedback I get from clients is that [greenspace programmes] are refreshing.* (Beth, Staff).

### Programme theory theme: individual self

#### Programme theory three: physical activity

Availability of resources, including trained staff and suitable equipment, were confirmed by the data as important contexts. Related to this, weather was mentioned by most interviewees as a context to plan for so that it does not negatively impact the programmes. The refinement of time on the programme as a contextual factor was confirmed by the consolidation interviews:

*The physical activities would have an extremely positive effect on people’s recovery outcomes, as long as they were engaged for a good period of time.* (Harry, Staff).

Interviewees agreed that enjoyment of activities was the key mechanism within this programme theory. Increased engagement with physical activity was established as the main short-term outcome; even if this was not high intensity exercise, the client was “*getting outside, getting fresh air, moving around”* (Gemma, Stakeholder). Improved positive mental health and improved physical health were confirmed as longer-term outcomes, for example through improving immune function and strength.

#### Programme theory four: self-efficacy

Availability of competent facilitators was confirmed by the data to be a key contextual factor. However, consolidation interviews also identified the need for a supportive and safe learning environment where participants were able to feel more confident about trying new activities. Time on the programme was confirmed as essential during consolidation interviews, again recognising that short-term funding could make longer programmes difficult. The mechanisms in this programme theory were confirmed to be empowerment and the *“huge impact on confidence”* (Gemma, Stakeholder) from learning skills, or re-learning old skills. Self-esteem was previously identified as an outcome in stage one interviews, but consolidation interviews highlighted that this was more accurately described as a mechanism. As a result of increased empowerment, confidence, and self-esteem, the main outcome in this programme theory was agreed to be increased application of skills to the lives of clients outside of programmes:

*They are given transferable skills and a sense of capacity to emotionally regulate in a different way […] There is something about working in that natural framework that helps you learn to cope with loss and helps you learn to cope with failure, because it’s part of the process.* (Beth, Staff).

#### Programme theory five: having a purpose

The structured routine of the programme was confirmed to be a key contextual factor. Consolidation interviews highlighted the need for programmes to have a person-centred focus, and not assume a one size fits all approach. Feelings of purpose was confirmed as a key mechanism, and interviewees spoke about how this was apparent across a range of different activities and programmes. Linked to feelings of purpose were positive changes in self-identity:

*There is always a sense that activities are about distraction, but it’s about meaningful and purposeful engagement. […] They then don’t just identify as someone who self-harms or uses substances, they identify as someone who can use a set of loppers, and remove some rhododendron, and plant a tree that will grow and serve a benefit. (Beth, Staff).*

The main outcome in this programme theory was agreed by interviewees to be improved self-esteem. This was said to often have an impact on a client’s future plans. However, as identified in stage one stakeholder interviews, the goal of employment or volunteering will not be a goal for everyone:

*For some people we have to accept that the programme is sort of the main goal for them. […] If we said “right okay, we haven’t got space for you anymore” it could be extremely detrimental to their recovery.* (Harry, Staff).

### Programme theory theme: social self

#### Programme theory six: relationships with facilitators

Programmes having a ‘doing with’ culture was confirmed by the data as a necessary contextual factor with one staff member saying that if clients were simply told what to do, rather than facilitators interacting alongside them, then this held little value. The need for facilitators to work in a trauma-informed way was also confirmed as key, especially relative to knowing how a person’s experiences may impact their engagement and relationships. Diversity of facilitators on the programme was also confirmed as important:

*There is less engagement from some people, Black and Asian communities for example, and some of that is thought to be due to lack of role models.* (Annie, Stakeholder).

The need for clients to have enough time on the programme was described as essential since the longer they were on the programme the higher the likelihood of building relationships with facilitators and subsequently engaging with the programme. Within these contexts, it was confirmed that decreased power imbalance and feelings of trust and safety with facilitators were central mechanisms. One interviewee described the reduction of “*power plays”* (Harry, Staff) in greenspace programmes, compared to clinical services, as fundamental. Communication was also confirmed as an important mechanism, with one interviewee explaining that the success of programmes was *“based around good conversations”* (Richard, Stakeholder). Similarly, feeling respected was identified as a mechanism, which links closely to the other mechanisms in this theory. In turn, these mechanisms reportedly lead to increased engagement from clients since “*clients engage and buy into programmes when it’s being provided by someone they know and trust”* (Annie, Stakeholder).

#### Programme theory seven: increased communication through shared experiences

Greenspace acting as an enabling environment was confirmed in consolidation interviews as a necessary context, with one staff member describing it as a “*real life setting”* (Harry, Staff), compared to more clinical settings. Engagement of others in the group was also confirmed as important, with one stakeholder saying that, from their experience, clients found it easier take part when their peers were also there. The availability of trained facilitators was also deemed essential with leadership being needed “*on the ground”* to aid group interactions (Gemma, Stakeholder). Increased communication through shared experiences was agreed as the central mechanism, with one stakeholder explaining, *“sharing an activity breaks down barriers”* (Gemma, Stakeholder). The increase in communication was confirmed to allow clients to “*foster relationships again”.* (Beth, Staff).

#### Programme theory eight: reduced isolation

The greenspace as an enabling environment, engagement of peers, and availability of trained facilitators to support client interactions, were confirmed to be the contexts linked to this programme theory, as well as the theory above. Under these contexts, mechanisms were agreed to be increased understanding of others, and reduced judgement and stigma. One staff member spoke about how the conservation work being done on some programmes aided in reducing stigma from the wider community:

*The work that they are doing is being seen, it’s profiling for people in recovery, so it breaks down a lot of stigmas.* (Harry, Staff).

These mechanisms were confirmed to lead to reduced isolation and clients integrating and reconnecting back into their community.

### Programme theory theme: macro-level

#### Programme theory nine: COVID-19 impact

COVID-19 was confirmed to be an important macro-level contextual factor for greenspace programmes, with one staff member describing the pandemic as “*a real challenge”* (Harry, Staff). The impact that COVID-19 had on programmes reportedly lead to the mechanisms of reduced trust and hope for some clients since services were unable to provide the same level of support since services were unable to provide the same level of support due to “*layers of considerations with health and safety issues”* (Beth, Stakeholder):

*They would have been running one-to-four* [people] *maybe, and now they are having to run one-to-one sessions, so all of a sudden three quarters of the people can’t attend.* (Richard, Stakeholder).

This was confirmed by the data to lead to reduced mental wellbeing in clients, and reduced number of people on the programmes. However, although interviewees discussed the difficulty of maintaining existing client engagement, some also discussed how the pandemic could present an opportunity to increase acceptance and engagement with greenspace programmes more generally, if staff and programmes were able to adapt, given “*the inherent risk of being indoors”* (Beth, Stakeholder). Another interviewee added that, in their opinion, engagement could increase, because online sessions can reduce barriers relative to initial engagement, which some clients struggle with. Further, engagement could be positively affected, because people appear to have become more aware of the benefits of nature due to increased positive media coverage:

*All these different narratives about the need to be in nature during lockdown to maintain good emotional wellbeing […] does that create more of a sense that service managers and funders will start to recognise that the pandemic does instigate a need […] It seems like this is an opportunity, with COVID, we need to think differently.* (Beth, Stakeholder).

### Programme theory theme: Meso-level

#### Programme theory ten: intervention approach

The explicit focus of the programme was confirmed as the central context in this programme theory as it can help establish whether people can self-refer or not. This was said to be an important consideration for safeguarding the clients and staff:

*Thinking mostly about people with milder mental health issues, so anxiety, stress, social isolation, those sorts of issues, which can be supported by more informal green health activities. But we’ve also been engaging with people like [organisation] who see people with much more potentially serious mental health, addictions and so on. […] Can clients trust what they are going to get, is it an appropriate level of support for their mental health needs?* (Annie, Stakeholder).

Additionally, the existence of a “*multidisciplinary team”* (Beth, Staff), and the right expertise among facilitators, was confirmed as necessary. In these contexts, interviewees reported that clients felt more supported, the main mechanism in this programme theory. Feeling supported was said to lead to satisfaction with and commitment to individualised outcomes, with clients being described as *“much more likely to engage with the activity”* (Annie, Stakeholder).

#### Programme theory eleven: stakeholder buy-in

Continued availability of funding was confirmed to be a necessary context relative to buy-in, although the funding cycle was described as detrimental in convincing wider stakeholder buy-in:

*You’ve just built up some trust and relationships with the health professionals, and then the funding runs out, and you have to start all over again.* (Richard, Stakeholder).

The need for clear objectives and outcome measures was also described as key:

*How do you pull together a real meaningful framework for justifying the need for programmes like this and funding them? […] What are the activities that we can be undertaking, and what exactly is it that our service users, our participants, can gain from this?* (Beth, Stakeholder).

Stakeholder experiences of greenspace programmes was also confirmed as a context. One staff member spoke about how if wider stakeholders, such as GPs, have no personal experience of how greenspace programmes can be effective then, irrespective of funding availability, there might still be resistance. If funding is available, programme objectives and measures clear, and stakeholders have positive first-hand experience of time spent in greenspace, then this was said to facilitate the mechanism of belief that the programmes are worthwhile. In turn, this was confirmed to lead to increased stakeholder buy-in. This could be in the form of increased funding or increased referrals. Indeed, one interviewee spoke about how, if one GP bought into greenspace programmes, this can enable wider buy-in:

*It is passed on from prescriber to prescriber, so if one prescriber had a positive experience, they tell their colleagues, and they get in touch because they want to join.* (Gemma, Stakeholder).

### Final programme theories

Table 4 shows the final consolidated programme theories and corresponding CMOcs. These are presented as ‘if (context), then (outcome), because (mechanism)’ statements. Presenting findings as ‘if-then-because’ statements has been recommended to explicitly show causality between components, a central component of realist work [27].

**Table 4:** Final consolidated programme theories and corresponding CMOcs shown as ‘if-then-because’ statements

Programme Theory Theme	Programme Theory Name	CMOc shown as an ‘if-then-because’ statement
Nature	Escape and getting away	If there is easy access to a quality greenspace environment with a planned programme, then mental wellbeing will be improved and stress will be reduced, because of feelings of ‘being away’, being present, reduced rumination, feelings of awe, and a connection to nature.
Nature	Space to reflect	If there is greenspace to provide physical space and a neutral, non-clinical backdrop for therapeutic conversations then, as long as there is adequate time spent on the programme, this results in increased discussion and opening up, because clients no longer feel ‘boxed in’ and confined, and they have space to reflect.
Individual self	Physical activity	If there are a variety of activities available, and programmes have the right resources such as staff and equipment suitable for poor weather, and if clients have enough time on the programme, then this will lead to increased engagement and improved physical and mental health, because clients will enjoy the activities they do.
Individual self	Self-efficacy	If there are available, trained facilitators to lead programmes, and the programme environment is supportive, and if clients have enough time on the programme, then clients will learn new skills and be more confident in applying skills to their lives outside of the programme, because of increased feelings of empowerment and confidence from learning new skills or relearning old skills.
Individual self	Having a purpose	If a programme provides structure and routine and provides a person-centred focus, then the self-esteem of clients will increase, because of an increased sense of purpose and changes in self-identity.
Social self	Relationships with facilitators	If a programme has a ‘doing with’ and not ‘doing for’ culture, is trauma-informed, is of adequate length, and if facilitators are from a range of backgrounds, then clients are more like to engage with, and buy into programmes, because there is decreased power imbalance, increased communication and feelings of trust and safety, and clients feel respected.
Social self	Increased communication through shared experiences	If the greenspace programme provides an enabling environment, in comparison to typical treatment environments, and if there are trained facilitators to guide group dynamics and interactions with peers, then this leads to improved relationships with peers and others, because of increased communication through shared experiences.
Social self	Reduced isolation	If the greenspace programme provides an enabling environment, in comparison to typical treatment environments, and if there are trained facilitators to guide group dynamics, then isolation is reduced and clients integrate and ‘reconnect’ back into their community, because there is increased understanding of others and decreased stigma and judgement.
Macro-level	COVID-19 impact	If COVID-19 and related restrictions exist, then mental wellbeing is reduced, because programmes are unable to provide the same level of support and there is reduced trust of programmes and reduced feelings of hope for the future in clients.
Meso-level	Intervention approach	If programmes have an explicit focus and a multidisciplinary team approach consisting of the right expertise, then clients will feel satisfied with the programme and will be more likely to commit to the programme, because they feel adequately supported.
Meso-level	Stakeholder buy-in	If there is funding available to support the continuation of programmes, if programmes have clear objectives and outcome measures, and if wider stakeholders (such as funders or those signposting onto programmes) have experience or knowledge of the benefits of greenspace, then this will lead to stakeholder buy-in, because they will believe the programmes are worthwhile.

## Discussion

**Fig. 1:**
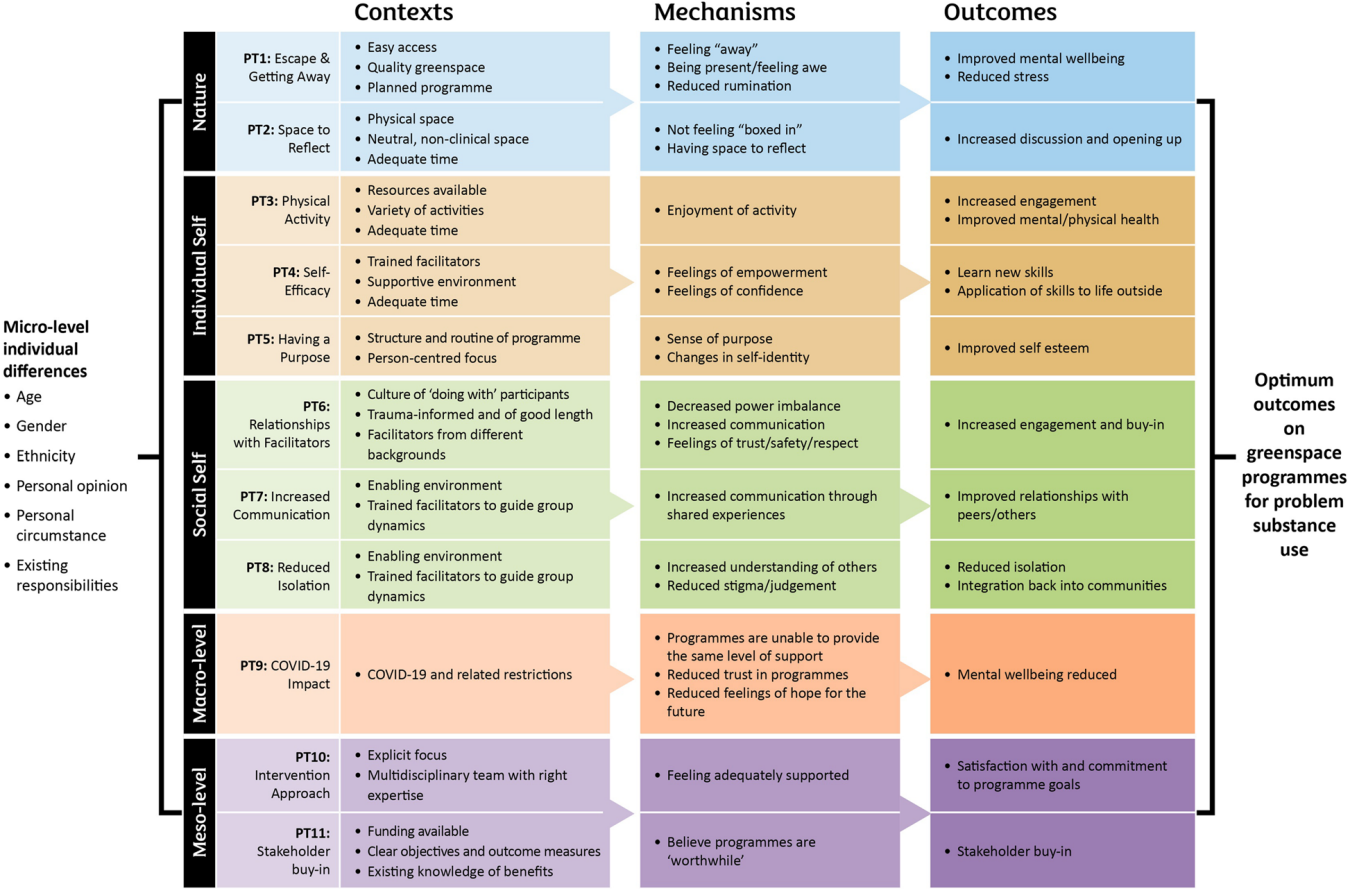
The refined model for greenspace programmes that support people with PSU

This study is a novel approach to understanding how greenspace programmes can be used to support people with PSU. Masterton et al. [12] originally identified seven programme theories which fell under three overarching themes of Nature, Individual Self, and Social Self. In this current study, while the original three theme headings of Nature, Individual Self, and Social Self still hold, eight refined and consolidated programme theories now fall under these headings. Additionally, data allowed identification of one macro-level programme theory relating to COVID-19, and two meso-level programme theories relating to stakeholder buy-in and intervention approach. Additional to the findings, and in line with social-ecological models, it is important to note that micro-level influences will also add an essential lens on how factors outside the programme may influence implementation and success [28, 29]. Based on this study’s findings, Fig. 1 shows an updated version of Masterton et al.’s original framework [12]. As well as additional programme theories, a key difference of this model is that it depicts the specific CMOcs that explain how greenspace programmes might be used to support people with PSU.

### Nature

This study’s findings suggest that time spent on greenspace programmes reduces stress. This is supported by literature across different domains, with research typically reporting at least short-term stress reduction as a key outcome when people visit greenspace [30–32]. Specific to people with PSU, horticultural therapy reduces self-reported stress in inpatient treatment programmes [16]. Aside from reduced stress, previous meta-analyses support the finding that connection to nature is increased through time spent in greenspace and is linked to improved mental wellbeing [33, 34]. However, this finding appears to vary between individuals, and some evidence suggests that visits to greenspace were only associated with higher wellbeing for individuals who initially felt less connected to nature [35, 36]. Various interviewees in the current study discussed the influence of prior experiences of nature, and how many clients with PSU on greenspace programmes frequently have limited experience of, and connection to, nature which could influence initial engagement. When drawing on findings such as those by Martin et al. [35] and Richardson et al. [36], it may be that this client group could benefit more than other groups who do have existing experience of nature, as long as clients are adequately supported initially and throughout the programme, and other barriers to access are addressed.

This study also indicates that time spent in greenspace allowed clients to feel removed from their daily lives. For people with PSU, this concept of removal to a different environment has been shown to be present in interventions for substance use such as wilderness therapy [37, 38]. In wilderness therapy programmes, an intrinsic feature of the intervention is immersion in nature and separating clients from their everyday lives and stressors, including family, social, and living environments [39], with clients describing this feeling of ‘getting away’ as essential in their support journey [40]. Additionally, the greenspace used on programmes was described in this study as neutral, in comparison to traditional treatment settings which are typically situated within organisational buildings inherently linked with statutory health services. This is important since mistrust of statutory health services is frequently cited among people with PSU [41, 42]. Part of this mistrust may be attributed to perceived stigma that can be attached to substance use treatment, whereas this is reportedly not as present in greenspace programmes [40]. Feeling less confined in a neutral space was said to lead to increased therapeutic conversations, but this process was not an instant process. Mistrust of health services can be substantial for people who have been systemically marginalised and stigmatised, so clients need adequate time to build up trust with facilitators and programmes. This is supported by literature exploring more traditional substance use interventions where increased amount of time on programmes led to higher likelihood of positive health outcomes [43].

### Individual self

Greenspace programmes can enable changes within an individual which improve overall physical and mental wellbeing. Despite this, existing evidence is inconclusive regarding the relationship between greenspace and physical activity, with some studies supporting the association [44, 45], and others showing no relationship [46]. Previous studies have found that providing information about the need to undertake physical activity is likely not enough to change behaviour [47], and structured programmes are needed [1]. Indeed, having skilled and trained staff to initially support people on programmes was described as key in this study. As well as changes in physical activity, findings highlighted the potential for psychological changes, such as increases in self-efficacy, and feelings of purpose. Increases in self-efficacy can aid in coping with stressful circumstances and, for people with PSU, this could be particularly useful if they have previously found it difficult to manage challenges in their lives and are using drugs or alcohol as a way of dealing with trauma [48, 49]. In comparison to self-efficacy, findings relating to increased purpose showed how clients can gain a sense of purpose from activities that they do on greenspace programmes. A renewed sense of purpose has been reported as important in recovery-orientated, abstinence-based groups [50, 51]. However, this study’s findings suggest that this mechanism is not limited to abstinence-focused environments and appears central to harm reduction approaches too. A sense of purpose was linked to positive changes in self-identify which has also previously been identified as important so people with PSU feel less characterised solely by their substance use [52].

### Social self

Therapeutic relationships have been reported to account for as much variance in therapy outcomes as the treatment modality itself [53]. The crucial role of relationships in treatment and support has previously been discussed relative to greenspace programmes [54, 55], and is further evidenced in the current study. Some participants stated that improving relationships was the most important long-term outcome, over and above reducing substance use. One reason that building relationships was said to be better enabled in greenspace programmes was the removal of the traditional professional/client relationship and subsequent power imbalances. Berger [56] highlights the issue of power within the therapeutic process, proposing that, traditionally, the therapy space is designed, controlled, and owned by the therapist, which subsequently sets up unavoidable power imbalances. In PSU treatment specifically, power imbalances are well documented [57, 58]. However, service providers can be hesitant about challenging the status quo due to continuously reinforced beliefs that professional authority must be upheld [59, 60]. Conversely, greenspace environments are described as more democratic because the space is neither owned nor controlled by facilitator or client. As a result, a reduced power imbalance reportedly increases trust between clients and facilitators and allows clients to feel respected. To further increase client trust, findings suggested that programme culture should also be trauma-informed, with facilitators being knowledgeable of how clients’ lives and experiences impact their day-to-day interactions. Fernee et al. [61] discussed this context relative to wilderness therapy and highlighted that a trauma-informed way of working ensures a caring and non-confrontational approach. In turn, these contexts and mechanisms can lead to increased client engagement and buy-in.

As well as relationships with facilitators, the improved relationships with clients’ peers on the programme was discussed at length. Research has shown that greenspace programmes often provide situations where peer support is encouraged through challenging tasks, promoting dialogue in a way that rarely happens in other types of treatment/health services [40, 62]. Evidence exists that suggests positive peer relationships can successfully support people with PSU [53, 63], including supporting reductions in use [64]. Further, recent research identified that positive peer relationships and reduced substance use is more strongly associated in greener environments [65]. Additional mechanisms which were said to facilitate improved relationships were feelings of acceptance, belonging, and a reduction in perceived stigma. While feelings of acceptance and reduced stigma have previously been reported on greenspace programmes [55, 62, 66], this study suggests that these mechanisms are particularly important for people with PSU who often experience higher levels of stigma compared to those with other mental health challenges [67]. Stigma has been shown to be associated with maintaining PSU, increasing the likelihood of drug and alcohol related harm, and reducing the likelihood of accessing support services [53, 68–70]. Therefore, a reduction in stigma is likely a critical mechanism for this client group in achieving positive substance use outcomes if that is their goal.

### Macro-, meso-, and micro-level considerations

At a macro-level, the COVID-19 pandemic had various negative effects on programmes. Interviewees mentioned that clients appeared to lose trust in programmes due to closures, unreliability, and unpredictability, and they also reported increased feelings of hopelessness because of the pandemic. These findings have been shown in other studies exploring the effect of the pandemic on services for people with PSU as it brought a period of intense disruption, isolation, and confusion to many people who were reliant on services for support [71]. Despite this, opportunities may also be presented by the pandemic. Some participants in this study spoke about how increased mental health challenges as a result of the pandemic may increase footfall, particularly given the increased focus on, and awareness of, the benefits of nature through periods of lockdown. However, services must address and adapt to changing contexts in order to be dependable and stable, key components of effective treatment [53, 71].

From a meso-level perspective, there should be an explicit and clearly communicated focus of the programme and a suitable multidisciplinary team approach. Some clients could benefit from a programme that provides holistic support but does not require a commitment to abstinence or a reduction in substance use. Other clients may specifically seek out programmes with an abstinence focus as part of their own recovery journey. Concern was raised by interviewees that if programme aims are not explicit then clients may have different expectations in comparison to what the programme is able to offer and thus feel unsupported and dissatisfied with outcomes. These findings are supported by a recent realist review of social prescribing engagement and adherence which discussed how people are much more likely to engage with a particular programme if it matches their expectations, and those with unrealistic expectations were least likely to maintain adherence [72]. While stakeholder buy-in was identified as another meso-level programme theory, this was also linked to wider macro-level contextual factors such as funding availability. The challenges created by lack of secure funding and uncertainty about future provision has been identified through recent green prescribing reports [3, 11]. Specific to PSU, uncertainty about funding for support services has also been well documented [53], and lack of secure funding dramatically reduces the perceived sustainability of programmes meaning stakeholders are less likely to buy-in to programmes. Having clear objectives of programmes and explicit outcome measures were suggested as a way of mitigating this by increasing positive views of programmes.

Despite this study showing that greenspace programmes can be successful in supporting people with PSU, it is important to recognise that this is of course not a homogenous group, and micro-level individual characteristics and experiences will contribute to shaping programme success. Factors such as age, gender, ethnicity, and personal opinion, will likely impact programmes, but their heterogeneity means that it is very difficult to develop CMOcs that are generalisable across clients. However, in realist research a level of pragmatism must be adhered to, given that there could potentially be infinite numbers of CMOcs. By identifying these individual level factors as currently unconfigured contextual factors, this acknowledges that they likely play a role in programme success but require further exploration, possibly on a case-by-case basis.

### Policy and practice implications

While the findings of this study support continued development and implementation of greenspace programmes as a legitimate route to health, to truly incorporate greenspace programmes in current health care provision there is a need for sustained investment from wider stakeholders and more secure funding. There is also a need for more effective partnership working between different sectors, with more advocacy, peer support, and training accessibility across all sectors involved [4, 73]. There should be clear guidance and parameters for programme development, with programmes being explicit about who they are designed to support [74]. There must be awareness that greenspace programmes may exacerbate inequalities if clients’ needs are not considered central to the programme, and there should be awareness of programmes being implemented more readily in certain areas than others which can negatively impact accessibility for those potentially most in need of support [4].

Although greenspace programmes exist for people with PSU, until this point there has been no framework showing why they are successful and in what contexts. Without this knowledge it is difficult to successfully replicate and implement new programmes. What this study has shown is that many of the key components that make greenspace programmes successful for people with PSU are also seen across other typical treatment pathways. For example, individual-level changes, such as increases in self-efficacy and feelings of purpose, as well as social-level changes, such as improvements in relationships, have previously been identified as mechanisms in holistic PSU treatment [53]. What greenspace programmes add to this is the therapeutic effect of immersion in nature, and how this allows clients to feel that they are ‘getting away’ from their own lives and daily stressors, giving them ‘space to reflect’. Further, increased levels of physical activity can contribute to both physical and mental health. Greenspace programmes are also flexible in a way that traditional treatments are often not. For example, they can provide support without falling under the typical banner of ‘treatment’, and this can reduce stigmatisation. The ability of greenspace programmes to support people without pre-existing requirements and eligibility criteria indicates that they could be a beneficial addition to a package of holistic care. They are also often less expensive to provide than other interventions [75].

Finally, addressing substance use needs effectively and holistically has the potential to lead to significant public health impact and improve population health, particularly among those already facing health and social inequalities. In Scotland specifically, the high rates of PSU and substance-related harm [76, 77] suggest that novel approaches to treatment and/or support, such as greenspace programmes, could be a beneficial addition to social care. Priorities set out by the Scottish Government priorities [78], as well as recommendations suggested by the Drug Deaths Taskforce [79] and regional panels such as the Dundee Drugs Commission [80] highlight that tackling stigma; delivering a whole systems model of care; an increased focus on engaging with those who do not currently access services; and additional funding for community-based projects are all essential in mental health and substance use treatment and support going forward. All of these aspects have been cited as central components of greenspace programmes.

### Limitations and future research required

It is essential to acknowledge that greenspace programmes are not a ‘silver bullet’. There are situations where greenspace programmes may be unsuitable for clients, and circumstances where they will have very little effect. For example, people with PSU can experience wider vulnerabilities and face systemic challenges such as marginalisation, trauma, insecure housing, and entrenched poverty that result in continuing inequalities [81]. Greenspace programmes were described by one interviewee in this study as a ‘drop in the ocean’ when acknowledging the wider, structurally violent landscape that people with PSU often experience [82]. Although this study has highlighted the potential of greenspace programmes providing aspects of care that other approaches do not, the limitations of what greenspace programmes provide must be made clear and should not be oversold.

For the framework to truly represent the CMOcs through which programmes are successful, client voice should be incorporated in future work. Future work must also consider how best to measure outcomes so that this component of the realist framework is more detailed. The updated MRC/NIHR framework for developing and evaluating complex interventions [83] describes the choice of outcome measures as a *“crucial aspect”* (p.7) in intervention and evaluation design, and consideration must be given to which outcome measures to include, and how best to navigate multiple outcomes at an individual- and/or system-level. Relative to implementation and evaluation of greenspace programmes, future work should also look to incorporate more quantitative outcome measures, something supported by existing evidence [3, 84, 85]. For example, the use of validated psychometric assessment tools and/or physiological measures could allow a deeper understanding of how greenspace programmes can support people with PSU [84, 85].

## Conclusion

With high levels of substance-related harm in Scotland, novel approaches to addressing this public health emergency should be considered. Greenspace programmes may effectively support people with PSU as they can provide holistic support without requiring strict criteria, such as reducing or stopping substance use, to be met, therefore reducing barriers to treatment and support. Greenspace programmes have previously been shown to support mental health due to: feelings of escape; space to reflect; physical activity; self-efficacy; feelings of purpose; relationships with facilitators; and shared experiences [12]. Interview data in this study supported Masterton et al.’s framework for use on programmes that support people with PSU but also identified that programmes must additionally consider: explicit intervention focus to ensure adequate support; existing challenges with funding and stakeholder buy-in; and the impact of COVID-19. The findings of this study are theoretically novel and have practical relevance for both policymakers, and those designing such interventions, by providing insight into how best to optimise, tailor, and implement future greenspace programmes for PSU support.

## Supplementary Information

**Additional file 1.** Interview schedule. The interview schedule used to collect data in the qualitative interviews.

## Data Availability

The datasets generated and/or analysed during the current study are not publicly available due to individual privacy rights and individual privacy being compromised if the dataset is shared due to the small sample included.

## Abbreviations

CMOc: Context-mechanism-outcome configuration
CMO: Context, mechanism, outcome
ESRC: Economic and Social Research Council
GUEP: General University Ethics Panel
GP: General practitioner
MRC/NIHR: Medical Research Council/National Institute for Health and Care Research
MS Teams: Microsoft Teams
N/A: Not applicable
NHS: National Health Service
PSU: Problem substance use
UK: United Kingdom
