# Supplementary material for: Exploring how greenspace programmes might be effective in supporting people with problem substance use: a realist interview study
Source: BMC Public Health. 2022 Sep 1;22:1661. doi: 10.1186/s12889-022-14063-2 (PMC9436451; doi:10.1186/s12889-022-14063-2)
Supplement: Supplementary file 1 — Additional file 1. Interview schedule. The interview schedule used to collect data in the qualitative interviews. [file 12889_2022_14063_MOESM1_ESM.docx]

**Additional File 1: Interview schedule**

Black text = stage one and stage two interview questions

Blue text = refined and added questions in stage two only

**1. Can you give me a brief overview of your role?**

**OUTCOMES**

**2. What do you consider to be the main desired outcomes/goals on greenspace programmes for mental health and PSU?**

*Probes*

Do you think that desired outcomes/results are the same for all people on the programme?

In what ways are they different?

What differences are there in outcomes for gender/age/ethnicity/different MH diagnoses?

**3. How do you measure mental health/substance use outcomes/results in your service?**

*Probes*

Could they be measured any differently?

How do you think outcomes could best be measured on a new programme?

**4. Some programmes offer wider support, and some are more targeted. As soon as it is labelled a ‘mental health programme’ or ‘substance use support’, it can become medicalised. How might the programme label have an effect?**

*Probes*

Do you think programmes should specify intended outcomes?

Do you think there are challenges around that?

**MECHANISMS**

**5. What are the most important aspects of the programme for mental health?**

**6. What are the most important aspects of the programme supporting people with PSU?**

**You have spoken about the outcomes of programmes, and I am really interested in how these outcomes are achieved. We have identified some mechanisms by which we believe programmes to be successful and so I was wondering if you could discuss your opinion on some of these. There is no right or wrong answer so feel free to disagree or expand on any section.**

**7. Do you think that greenspace programmes allow participants the feeling of getting away and the feeling of being removed from their everyday lives and stressors?**

*Probes*

What kind of feelings do you think the green environment evokes in participants?

What do you think it is about the greenspace environment that is beneficial?

**8. Do you believe the participants can use the greenspace as a space for reflection?**

*Probes*

What are your thoughts on participants feeling that they are not closed in by four walls as in traditional mental health or substance use services?

**9. Do you think that the programmes increase a connection to nature?**

*Probes*

Do you think that the connection to nature influences participants?

Do you think it is harder for participants who have had no previous experience with being in nature to engage with programmes?

**10. Physical activity is often part of greenspace programmes, but what needs to be in place for physical activity to happen and to be appealing to participants?**

*Probes*

How important is it to have trained facilitators to lead sessions?

Do you think it is important to offer a range of activities?

Do you think there are barriers to physical activity? For example, weather, discomfort, physical challenges? How could they be addressed?

**11. Do you think that the participants can learn both physical and psychological skills on the programme, and can you give examples? (e.g. self regulation, coping with challenges, social skills)**

*Probes*

How are new skills helpful outside the programme in their own lives?

**12. Feelings of responsibility and purpose appear to increase in many greenspace programmes, how do you think that carries into their lives outside the programme?**

*Probes*

Do you think that the routine of the programmes is helpful?

**13. What is it about relationships with facilitators that is important on greenspace programmes?**

*Probes*

Participants may have had negative experiences before with healthcare providers or have experienced failures with safeguarding, and this can lead to challenges with trust. How best can that be addressed to encourage participants to take part?

How can programme facilitators actively reduce the power inequality and promote empowerment?

How can programme facilitators best support people at the end of the programme?

**14. How do greenspace programmes allow increases in social skills and reduced isolation?**

*Probes*

Do you think that greenspace programmes can help reduce stigma within the group, and how?

How do you think other group members can influence engagement? How can this be managed?

Do you think there should be a range of ages and expertise within a group?

**CONTEXTS**

**15. We’ve seen that greenspace programmes work differently in different places. What is it about the way one organisation works compared to another organisations that makes a difference to implementation?**

*Probes*

Location, funding, size of group, staffing, client groups, quantity or quality of greenspace

**16. In your opinion, what is a ‘quality’ greenspace, what aspects do you think are crucial within a space to promote or encourage use?**

*Probes*

What facilities e.g. toilets are needed?

What about lighting, seating areas, level paths?

Do you think people prefer biodiverse green spaces?

**17. Could you talk about how you think the existing view of greenspace programmes by primary care professionals impacts the success of programmes?**

*Probes*

Do you think those in primary care accept greenspace programmes as effective for health care?

Do you think it is easy to convince stakeholders of their worth?

How can stakeholders be convinced to buy-in?

Does that affect funding?

How could programmes be better promoted?

**18. What are the other challenges or barriers in the success of the programme?**

*Probes*

Are programmes successful for everyone? Are there limitations?

Have you seen any challenges with access to programmes, is accessibility and/or transport an issue?

**19. If you could change something about a programme to make it work more effectively, what would you change and why?**

**20. How has Covid-19 affected greenspace programmes?**

**FINAL QUESTION**

**21. What else do you think we need to know, to really understand how this programme has worked here?**
